# Research interrupted: applying the CONSERVE 2021 Statement to a randomized trial of rehabilitation during critical illness affected by the COVID-19 pandemic

**DOI:** 10.1186/s13063-022-06640-y

**Published:** 2022-09-02

**Authors:** Julie C. Reid, Alex Molloy, Geoff Strong, Laurel Kelly, Heather O’Grady, Deborah Cook, Patrick M. Archambault, Ian Ball, Sue Berney, Karen E. A. Burns, Frederick D’Aragon, Erick Duan, Shane W. English, François Lamontagne, Amy M. Pastva, Bram Rochwerg, Andrew J. E. Seely, Karim Serri, Jennifer L. Y. Tsang, Avelino C. Verceles, Brenda Reeve, Alison Fox-Robichaud, John Muscedere, Margaret Herridge, Lehana Thabane, Michelle E. Kho, on behalf of the CYCLE Investigators

**Affiliations:** 1 St. Joseph’s Healthcare Hamilton, Hamilton, ON Canada; 2 School of Rehabilitation Science, McMaster University, Hamilton, ON Canada; 3 Department of Medicine, McMaster University, Hamilton, ON Canada; 4 Department of Health Research Methods, Evidence and Impact, McMaster University, Hamilton, ON Canada; 5 Division of Critical Care Medicine, Department of Anesthesiology and Critical Care Medicine, Faculty of Medicine, Université Laval, Quebec, QC Canada; 6 Department of Family Medicine and Emergency Medicine, Université Laval, Quebec, QC Canada; 7 Department of Medicine and Department of Epidemiology and Biostatistics, Western University, London, ON Canada; 8 Department of Physiotherapy, Austin Health, Heidelberg, VIC Australia; 9 Department of Physiotherapy, The University of Melbourne, Parkville, VIC Australia; 10 Li Sha King Knowledge Institute, St. Michael’s Hospital, Toronto, ON Canada; 11 Interdepartmental Division of Critical Care, University of Toronto, Toronto, ON Canada; 12 Department of Medicine, Faculty of Medicine and Health Sciences, Université de Sherbrooke, Sherbrooke, QC Canada; 13 Department of Anesthesiology, Faculty of Medicine and Health Sciences, Université de Sherbrooke, Sherbrooke, QC Canada; 14 Centre de recherche du CHI de Sherbrooke, Sherbrooke, QC Canada; 15 Division of Critical Care Medicine, Niagara Health, St. Catharines, ON Canada; 16 Department of Medicine (Critical Care), University of Ottawa, Ottawa, ON Canada; 17 School of Epidemiology and Public Health, University of Ottawa, Ottawa, ON Canada; 18 Ottawa Hospital Research Institute, University of Ottawa, Ottawa, ON Canada; 19 Departments of Medicine, Orthopedic Surgery and Population Health Sciences, Duke University School of Medicine, Durham, NC USA; 20 Department of Medicine, Hôpital Sacré-Coeur de Montréal, Université de Montréal, Montreal, QC Canada; 21 Department of Medicine, University of Maryland Medical Centre, Midtown Campus, Baltimore, MD USA; 22 Division of Pulmonary and Critical Care Medicine, University of Maryland School of Medicine, Baltimore, MD USA; 23 Department of Medicine, Brantford General Hospital, Brantford, ON Canada; 24 Department of Critical Care Medicine, Queen’s University, Kingston, ON Canada; 25 Toronto General Research Institute, University Health Network, Toronto, ON Canada; 26 Research Institute of St. Joseph’s, Hamilton, ON Canada

**Keywords:** Research report, Research design, Research personnel, Randomized controlled trials, Critical illness

## Abstract

**Rationale:**

The COVID-19 pandemic disrupted non-COVID critical care trials globally as intensive care units (ICUs) prioritized patient care and COVID-specific research. The international randomized controlled trial CYCLE (*C*ritical Care C*yc*ling to Improve *L*ower *E*xtremity Strength) was forced to halt recruitment at all sites in March 2020, creating immediate challenges. We applied the CONSERVE (CONSORT and SPIRIT Extension for RCTs Revised in Extenuating Circumstance) statement as a framework to report the impact of the pandemic on CYCLE and describe our mitigation approaches.

**Methods:**

On March 23, 2020, the CYCLE Methods Centre distributed a standardized email to determine the number of patients still in-hospital and those requiring imminent 90-day endpoint assessments. We assessed protocol fidelity by documenting attempts to provide the in-hospital randomized intervention (cycling or routine physiotherapy) and collect the primary outcome (physical function 3-days post-ICU discharge) and 90-day outcomes. We advised sites to prioritize data for the study’s primary outcome. We sought feedback on pandemic barriers related to trial procedures.

**Results:**

Our main Methods Centre mitigation strategies included identifying patients at risk for protocol deviations, communicating early and frequently with sites, developing standardized internal tools focused on high-risk points in the protocol for monitoring patient progress, data entry, and validation, and providing guidance to conduct some research activities remotely. For study sites, our strategies included determining how institutional pandemic research policies applied to CYCLE, communicating with the Methods Centre about capacity to continue any part of the research, and developing contingency plans to ensure the protocol was delivered as intended. From 15 active sites (12 Canada, 2 US, 1 Australia), 5 patients were still receiving the study intervention in ICUs, 6 required primary outcomes, and 17 required 90-day assessments. With these mitigation strategies, we attempted 100% of ICU interventions, 83% of primary outcomes, and 100% of 90-day assessments per our protocol.

**Conclusions:**

We retained all enrolled patients with minimal missing data using several time-sensitive strategies. Although CONSERVE recommends reporting only major modifications incurred by extenuating circumstances, we suggest that it also provides a helpful framework for reporting mitigation strategies with the goal of improving research transparency and trial management.

**Trial registration:**

NCT03471247. Registered on March 20, 2018.

**Supplementary Information:**

The online version contains supplementary material available at 10.1186/s13063-022-06640-y.

## Background

The novel coronavirus, SARS-CoV-2, first identified in Wuhan, China, in late December 2019 led to the World Health Organization (WHO) declaring a global pandemic on March 11, 2020. Concerns about personal protective equipment (PPE) supply and already strained health human resources challenged healthcare systems to minimize the demands on available resources, thus impacting the ability to conduct non-COVID-related research with patients. The anticipation of sharp increases in COVID-19 cases and hospitalizations raised further concerns about the availability and capacity to care for increased numbers of patients, the need for redeployment of research staff to meet clinical needs and/or prioritize COVID-related research, and mandates to transition to remote work.

COVID-19 imposed extenuating circumstances on clinical trials beyond the control of study investigators, sponsors, or funders. The CONSERVE (CONSORT and SPIRIT Extension for RCTs Revised in Extenuating Circumstance) 2021 statement was developed to extend the CONSORT (Consolidated Standards of Reporting Trials) [1, 2] and SPIRIT (Standard Protocol Items: Recommendations for Interventional Trials) [3] reporting guidelines to ensure the quality, completeness, and transparency of important protocol modifications due to extenuating circumstances for trials and trial protocols [4]. This guidance document encourages the research community to report how extenuating circumstances were managed, examine their overall impact, and take these modifications into account when interpreting trial results. Herein we report use of the CONSERVE-CONSORT Extension [4] as a framework to describe the CYCLE RCT (*C*ritical Care C*yc*ling to Improve *L*ower *E*xtremity Strength; NCT03471247) study management during the first seven months of the pandemic. The CYCLE trial is currently underway.

**Fig. 1:**
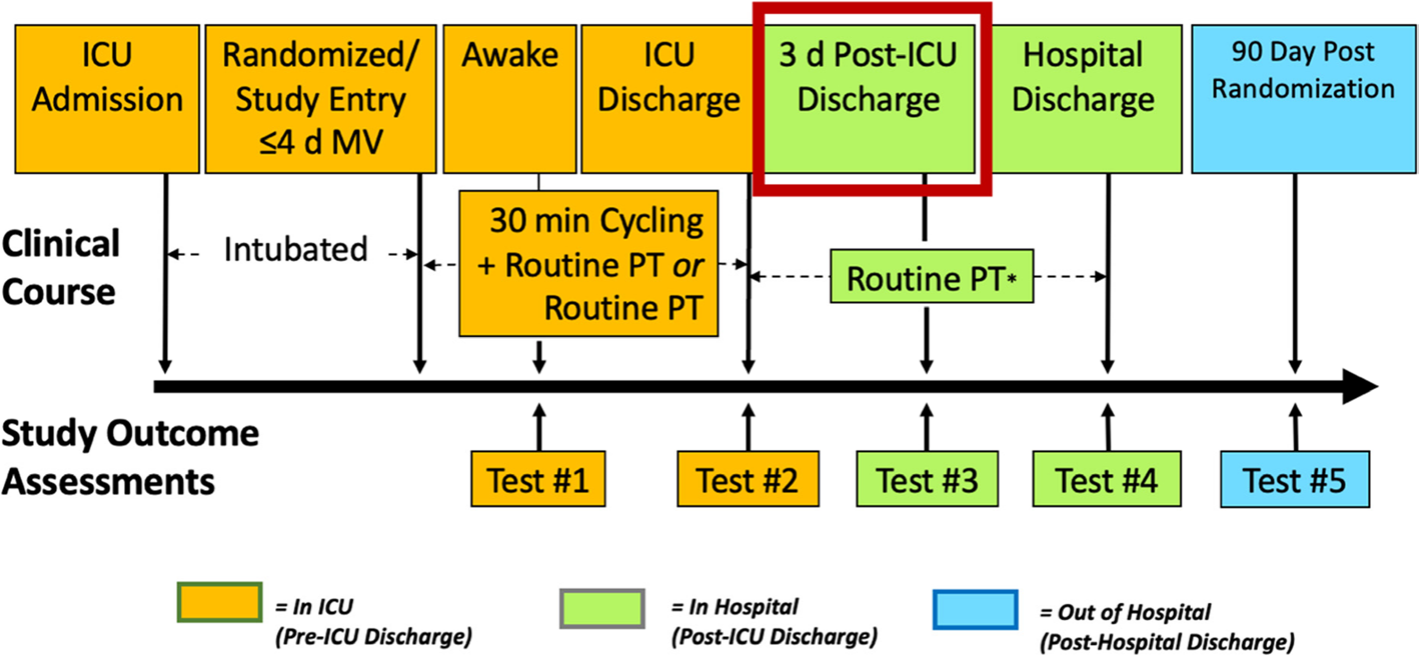
CYCLE study schema

CYCLE is an international multicenter randomized controlled trial (RCT) examining in-bed cycling in critically ill, mechanically ventilated adults (target *N* = 360). CYCLE involves a complex in-hospital rehabilitation intervention provided in the ICU and relies on a multidisciplinary team of frontline healthcare providers and research personnel to implement the protocol. Patients are randomized to either 30-minutes of daily in-bed cycling and routine physical rehabilitation (PR) or routine PR alone, delivered by ICU physical and occupational therapists (hereafter called “interventionists”) 5 days per week until ICU discharge or 28 days, whichever comes first. Physical outcome measures are administered at 4 time points through the index ICU and hospital admission; the primary outcome is the Physical Function ICU Test (scored) [5], a performance-based physical function measure assessed at 3-days post-ICU discharge by acute care therapists (physical and occupational therapists, and therapy assistants, hereafter called “assessors”) blinded to treatment allocation. Patient-reported outcomes, administered by research coordinators (RCs), are assessed at 3 time points through the index ICU and hospital admission and at 90-days post-randomization. Figure 1 outlines the study schema. Since study initiation in 2016, CYCLE has been enrolling patients in 17 sites in 3 countries (Canada, USA, Australia) and 2 languages (English and French). Further information about the protocol is located at https://clinicaltrials.gov/ct2/show/NCT03471247, and a protocol paper is in preparation. The CYCLE Methods Centre, comprised of the Principal Investigator, lead research coordinators, and research assistants, is based at St. Joseph’s Healthcare (Hamilton, Canada), the primary CYCLE study site.

With the global pandemic declaration, CYCLE recruitment was interrupted. At the time of the interruption, the CYCLE trial was actively enrolling patients and conducting follow-up assessments. The Methods Centre personnel were also preparing for a pre-planned 180-patient interim analysis (half of the target 360-patient enrollment). Immediately following interruption, the Methods Centre identified 3 main impacts to CYCLE’s internal validity, including (1) in-hospital randomized intervention delivery (cycling and routine PR), (2) obtaining in-hospital primary outcome data, and (3) accurate, timely data entry and validation for patient safety monitoring in preparation for the pending interim analysis. Our aims were to honor our research commitment to patients enrolled before the pandemic, optimize cohort retention, minimize missing data, and continue to conduct the trial where ever it was feasible and safe. This report describes the CYCLE trial management Methods Centre response to the pandemic up to September 30, 2020.

## Methods

The CYCLE Methods Centre developed several risk mitigation strategies to minimize impact of the COVID pandemic on the internal validity of the CYCLE RCT and report these using the framework of the CONSERVE-CONSORT Extension guidance [4]. The CONSERVE-CONSORT checklist can be found in Additional File 1: Appendix 1. To account for randomized patients (item 13: participant flow—number of participants randomized, losses and exclusions after randomization), we first developed structured communication using a standardized email (Additional File 1: Appendix 2) to determine (1) patient status and identify patients at risk of not receiving the allocated intervention, those pending primary outcome assessment, and 90-day assessments scheduled to occur by April 30, 2020, and (2) site staffing and availability (i.e., interventionists, assessors, and RCs for tracking patients, coordinating blinded outcomes assessments, and administering patient-reported outcome measures in-hospital and at the 90-day follow-up).

We developed internal Methods Centre communications to track patients, data progress, and personnel (research and clinical) across sites with the goals of providing support and guidance as required. We used these same communication strategies to reactivate sites when non-COVID-19 research resumed in some centers in late Spring 2020.

To track protocol fidelity, we focused on attempts to conduct study procedures, allowing us to discern pre-specified reasons for not completing a study procedure from reasons imposed by the pandemic. An “attempt” included any time an interventionist, assessor, or RC tried to deliver the intervention, collect the physical function or patient-reported assessments, or collect the 90-day assessments, as per study protocol. An attempt may or may not have resulted in successful conduct of the planned task. For example, if a patient was too sick to receive the assigned intervention, this was consistent with our protocol and therefore not considered a protocol deviation. If a task was not attempted due to pandemic-related factors (e.g., lack of staffing, PPE supply), we classified this as deviation from the initial protocol and documented specific reasons.

Our study focuses on four metrics we deemed critical to study internal validity. The first three related to participant flow (CONSERVE item 13). We documented the following attempts: in the hospital, randomized intervention delivery in the ICU and collection of the primary physical function outcome at 3 days following ICU discharge; post-hospital discharge, 90-day assessments scheduled up to April 30, 2020, within the prescribed timeframe (i.e., from 83–120 days post-randomization). We chose randomized intervention delivery as a measure of protocol fidelity and the primary outcome because of its highest importance among all other outcomes. We selected the April 30 timeline based on 2 factors: (1) initial reports planned for lockdowns of only 2-weeks duration and (2) allowing time for the Methods Centre to develop processes for ongoing remote data collection should lockdowns persist. The final metric, related to statistical methods (CONSERVE item 12), was timely data collection and entry to ensure completion of the interim analysis.

We analyzed all data descriptively and present figures where relevant.

## Results

**Fig. 2:**
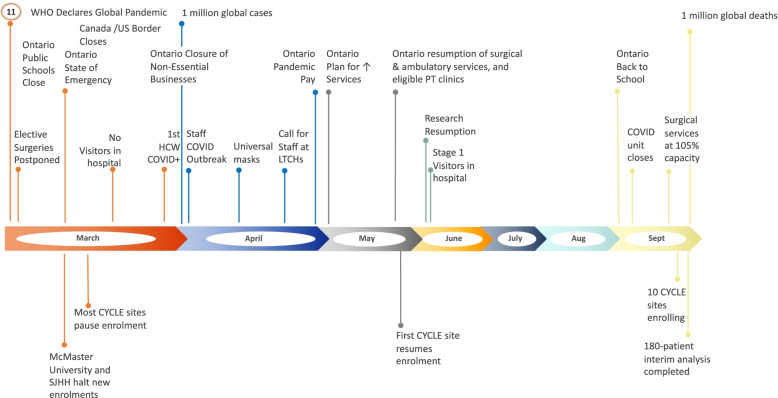
Timeline showing global, national, and CYCLE-related events during the COVID-19 pandemic from March-September 2020

By February 21, 2020, CYCLE had trained 17 sites and enrolled 50% of the 360-patient target, triggering the initiation of a planned 180-patient interim analysis. On March 17, 2020, the province of Ontario (home to the main research ethics board for CYCLE) declared a provincial state of emergency. At this time, 197 (54.7%) patients had been enrolled, and all actively enrolling sites (15 of 17 sites, including 12 academic and 3 community) were forced to pause recruitment into any non-COVID studies, including CYCLE. Figure 2 is a timeline of key events, both globally and related to the CYCLE trial. For patients already enrolled, study procedures continued according to each institution’s policy.

### Site characteristics

**Fig. 3:**
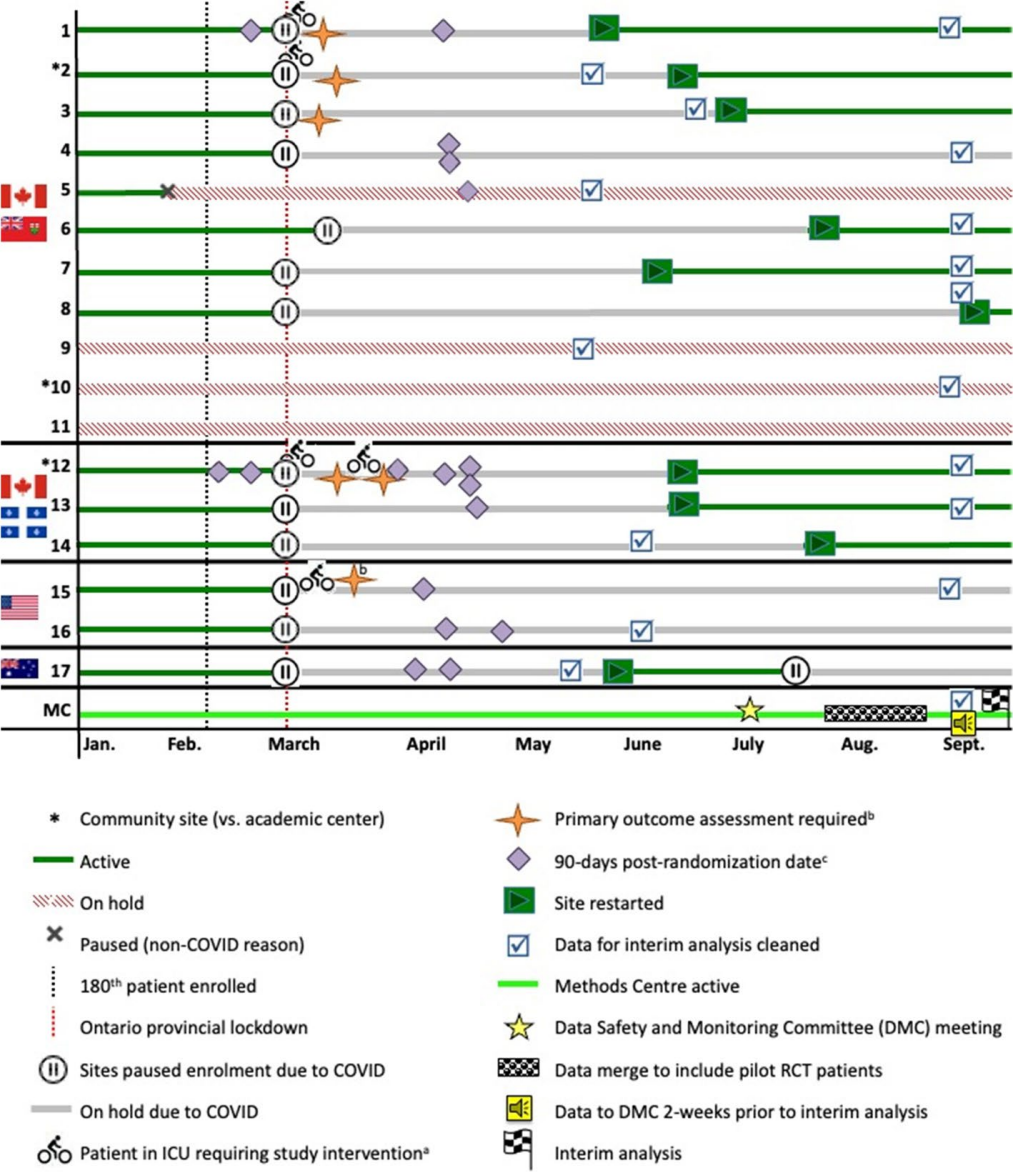
Timeline of CYCLE study sites from January to September 2020 with representation of patients of immediate concern during the first wave of COVID-19

Research personnel at all sites were required to work from home; at all but one site, interventionists and assessors were permitted to complete in-hospital activities for enrolled patients. Among the 15 active sites, we identified 26 unique patients (13.2% of total CYCLE cohort) still actively on the study protocol: 10 patients in hospital (5 patients still receiving study intervention in the ICU, 6 requiring primary outcome assessment), and 17 requiring 90-day follow-up assessments by April 30, 2020. Table 1 details progression through the protocol by site for these 26 patients. Figure 3 summarizes the activities of all CYCLE sites and the Methods Centre from January to September 30, 2020.

**Table 1:** Active patients and their study protocol status by site

Site location	Site	Patients in hospital^**a**^	Patients in ICU	Patients pending 3-day post-ICU Ax	Patients pending 90-day Ax by April 30, 2020	Unique patients
CAN - ON	1	2	1	1	0	2
	2	1	1	1	2	3
	3	1	0	1	0	1
	4	0	0	0	2	2
	5^b^	1	0	0	1	1
	6	0	0	0	0	0
	7	1	0	0	0	1
	8	0	0	0	0	0
	9^c^	0	0	0	0	0
	10^c^	0	0	0	0	0
	11^c^	0	0	0	0	0
CAN – QC	12	2	2	2	6	8
	13	1	0	0	1	2
	14	0	0	0	0	0
USA	15	1	1	1	1	2
	16	0	0	0	2	2
AUS	17	0	0	0	2	2
**TOTAL**		**10**	**5**	**6**	**17**	**26**

*Abbreviations*: *CAN* Canada, *ON* Ontario, *QC* Quebec, *USA* United States of America, *AUS* Australia, *ICU* intensive care unit, *Ax* assessment

^a^Patients in hospital *may* have already been discharged from ICU and had their 3-days post-ICU discharge assessment collected

^b^One patient had a prolonged stay in hospital, had completed the ICU interventions and primary outcome, but was pending their 90-day assessment

^c^Site not active at time of pandemic (paused for non-COVID reasons)

### Participant flow—intervention delivery

Five study patients were in ICU requiring the intervention; for all 5 patients, the allocated interventions were attempted at least once during the remainder of their respective intervention periods. Table 2 shows the number of attempted intervention days compared to the number completed, with reasons not done.

**Table 2:** Attempted versus completed tasks by intervention delivery in the ICU, primary outcome, and 90-day follow-up

	Patients (***n***)	Attempted (***n***/total, %)	Reasons not attempted	Completed (***n***/total, %)	Reasons not completed
**Randomized ICU intervention**					
**Routine PR**	5	15/15 (100%) days	N/A	12/15 (80%) days	Patient D/C from ICU before noon^b^ (*n* = 2) Therapist workload (*n* = 1)
**Cycling**	2	3/5 (60%) days	Research personnel not permitted on site^a^ (*n* = 2 days)	2/3 (67%) days	Patient D/C from ICU before noon^b^ (*n* = 1)
**Outcomes**					
3-days post-ICU physical function Ax*	6	5/6 (83%) Ax	Research personnel not permitted on site^a^ (*n* = 1)	3/5 (60%) Ax	Patient refusal (*n* = 1) Assessor perceived patient unable^c^ (*n* = 1)
**90-day Ax**	17	17/17 (100%) Ax	N/A	15/17 (88%) Ax	Unable to reach patient or SDM within timeframe (*n* = 2)

In this table, we summarize the attempted versus completed research activities and associated reasons. We highlight both pandemic and non-pandemic reasons for unsuccessful protocol delivery and outcomes assessments

*Abbreviations*: *PR* physical rehabilitation, *D/C* discharge, *ICU* intensive care unit, *Ax* assessment, *SDM* substitute decision maker

*Primary outcome for the trial

^a^Unable to attempt due to COVID reasons

^b^These reasons are within protocol and are not considered to be deviations

^c^Assessor perceives the patient is unable to complete an assessment due to safety concerns

### Participant flow—outcome assessments

Of the 10 study patients in hospital, 6 were pending primary outcome at 3-days post-ICU, and 5/6 (83%) were attempted. The primary outcome for one patient was not attempted due to an institutional policy prohibiting research staff from being on-site. Of 17 patients pending their 90-day follow-up assessments, 100% were attempted. Table 2 shows the number of attempted, successful, and missed outcomes, with reasons.

RCs identified 2 concerns for completing the 90-day follow-up assessments due to institutional directives to work remotely: (1) patient confidentiality (i.e., potential for identifying documentation in the homes of research personnel rather than in secured offices) and (2) research staff privacy (i.e., using personal phones to conduct follow-ups). In response, we developed a written guidance document to protect patient and research staff privacy and ensure data confidentiality (Additional file 1: Appendix 3).

### Statistical analysis—data entry and validation

For research staff working from home, the Methods Centre provided guidance for how to access and use the database remotely. The Methods Centre provided one-on-one support for data entry and cleaning. Working with sites, we validated data for the first 180-patients and the interim analysis was completed on-time in September 2020.

### Recruitment—site reactivation

**Fig. 4:**
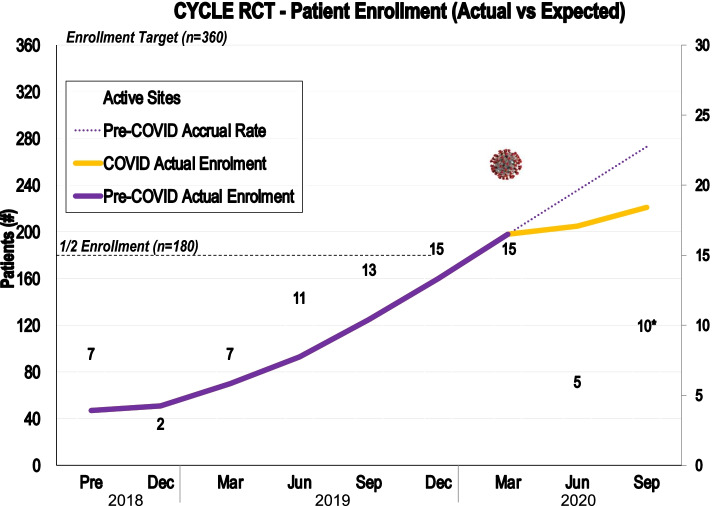
CYCLE RCT enrolment graph from start-up to end of September 2020. As of September 30, 2020, 221 patients had been enrolled accounting for 61% of the targeted enrollment

Using a similar communication strategy to the start of the pandemic, we developed a standardized reactivation email template that each site completed before resuming screening and enrolment (Additional file 1: Appendix 4). Through this template, we sought to ensure there was sufficient interventionist, assessor, and research personnel capacity to optimize protocol fidelity in the moment and through potential future waves. We also advised sites to exclude ICU patients with COVID-19 because of the unknown risks of disease transmission via bike equipment surfaces, strained therapy resources (i.e., due to increased clinical responsibilities to care for patients with COVID-19, or staff unable to work because of illness or quarantine), and PPE supply concerns. Figure 4 depicts the CYCLE enrollment graph from study initiation to the end of September 2020. By September 30, 2020, 10 sites (67%) had been reactivated, though recruitment was slower than pre-pandemic. With the commencement of the second wave in Fall 2020, 1 site was paused again at the direction of local leadership.

In Tables 3 and 4, we summarize key tips we learned for Methods Centres and study sites, respectively to optimize cohort retention under extenuating circumstances.

**Table 3:** Strategies for Methods Centres to address extenuating circumstances in an ongoing clinical trial

Strategy	Example from the CYCLE trial	Rationale
**1:** Identify patients immediately affected by the extenuating circumstances. Consider intervention delivery and safety, and primary outcome	Identified patients still in the ICU at highest risk for protocol deviations as they required the intervention, as well as those in hospital still requiring the primary outcome	Once high-risk points in the protocol were identified, site teams could develop strategies to mitigate missing data and losses to follow-up
**2:** Develop standardized communication tools to gather pertinent site information that is relevant to study conduct (e.g., staff capacity, patient status, data cleaning progress, etc.)	Developed structured email templates with focused questions regarding patient status and staff capacity	These templates allowed the Methods Centre to obtain precise information required to develop individualized strategies to promote protocol fidelity and retention
**3:** Develop standardized internal tools focused on the high-risk points in the protocol to facilitate real-time monitoring and timely identification of threats to study retention and fidelity	Revised existing tracking documents (e.g., data entry and validation to include a column indicating whether or not research personnel at each site has remote access to the database) to monitor and track patient progress by site	Tracking documents focused on high-risk points for patient status to mitigate missing data and losses to follow-up. Documenting the status of data entry and validation allowed the Methods Centre to continue to monitor the study for safety and protocol adherence and prepare for the interim analysis as scheduled
**4:** Continue as many trial activities as possible in preparation for future trial resumption at participating sites and the Methods Centre	Identified future trial milestones, such as data cleaning for the interim analysis, and future trial education. We worked with sites towards data entry, cleaning, and validation for the interim analysis. At the Methods Centre, we prepared for the interim analysis with our biostatistician for the data safety and monitoring committee. We modified in-person educational materials to virtual interactive sessions for future teaching	Maximizing the enrolment downtime provided opportunity to keep background work moving in a timely manner, and also facilitated ongoing Methods Centre personnel employment for study-related activities
**5:** Provide guidance for sites in preparation for trial resumption	Identified important considerations for protocol fidelity and cohort retention to help sites prepare to resume enrolment. We developed standardized communication tools for the site to prepare to restart research.	This allowed the Methods Centre to help sites optimize protocol fidelity in the context of uncertain future waves and potential resource challenges
**6:** Determine if major trial modifications are required or not required, considering current circumstances, and report rationale	Decision not to enroll patients with COVID-19 upon initial trial resumption due to infection control considerations for the equipment and strained therapy resources. We did not make major trial modifications	Reporting decision-making rationale will help readers assess the impact of the response to extenuating circumstances
**7:** Disseminate and share the lessons learned via peer-review publications	Collaborated with sites to carefully document the challenges and the solutions developed to mitigate them	Share best practices and experiences with the research community to inform future study management

**Table 4:** Strategies for Sites to address extenuating circumstances in an ongoing clinical trial

**Key strategies for sites to manage extenuating circumstances in an ongoing trial**	
**1:** Learn about institutional policies for research and assess how the policies apply to each study and circumstances. This knowledge will help the site and Methods Centre plan for how best to optimize retention and fidelity.	
**2:** Communicate with local trial stakeholders before restarting enrolment. Discuss with local study team members about what types of activities can still occur for a given study and the capacity of the site to continue with research within the confines of institutional policy and the current situation.	
**3:** Communicate with the Methods Centre regarding study-limiting institutional policies, site capacity to continue all or part of a research study, patient status, and data cleaning progress. Early and intentional communication will allow the Methods Centres to provide appropriate guidance and support for each site’s specific circumstances.	
**4:** Use materials developed for the extenuating circumstances to develop strategies for ongoing trial conduct. For complex studies involving multiple personnel, it is helpful to know staff availability to ensure the protocol is delivered as intended. Much can be learned from times of crisis and contingency plans are good practice, even outside pandemic times.	

## Discussion

Optimizing participant flow, cohort retention, and continuing with timely data entry and validation while assuring the safety of patients, interventionists, assessors, and research personnel were our primary goals in navigating the CYCLE trial through the COVID-19 pandemic. Our implementation priorities were driven by patients’ progress in the protocol at the beginning of the pandemic. Throughout the COVID-19 pandemic, we learned important lessons and implemented crucial processes to ensure research integrity, and patient and staff safety. In this report, we documented our approaches to address extenuating circumstances and patient retention for a complex rehabilitation intervention in critically ill patients requiring multiple personnel with distinct research and clinical roles, which will enrich the interpretation of the trial results. Moreover, lessons learned may help Methods Centres and sites navigate extenuating circumstances, such as the COVID-19 pandemic. In addition, these strategies may support future capacity-building efforts in rehabilitation science and enhance the rigor and quality of clinical trials outside of extenuating circumstances.

At the start of the pandemic, researchers expressed many concerns about the conduct of non-COVID-19 clinical trials. Members of our group collaboratively developed guidelines for continuing or restarting non-pandemic focused research [6] as many clinical trials were interrupted or halted by the pandemic [7]. A myriad of challenges from these interruptions have included lost treatment opportunities for patients [8, 9], threatened trial equipment supply chains [9], missed medication doses in vulnerable populations [10], uncertainties about re-starting trials [11], and challenges with data integrity and interpretability due to intercurrent complications (e.g., unavailable study drug, treatment discontinuation due to COVID-19 illness, and missing data) [12–14]. Few publications have described specific trial experiences in the context of the current pandemic. Shiely et al. reported management of 8 commercial clinical trials of investigational medicinal products during COVID-19 [15], underscoring challenges related to site communication, intervention delivery, participant retention, and data collection. For example, these investigators implemented protocol modifications to optimize intervention delivery away from in-hospital to home settings (e.g., to administer medication infusions to immunocompromised patients), and outcome assessments from in-person to telephone follow-ups. The authors also shifted from paper to electronic case report forms to facilitate remote collection and entry. However, not all trial interventions or outcomes assessments can pivot to virtual platforms or in-home visits, either due to the intervention and outcomes themselves, or the target population; for example, in-bed cycling with critically ill patients must be delivered in-person in the ICU and performance-based outcome measures cannot occur virtually.

Changing the modes of intervention delivery and outcome assessment may affect the internal validity of the study. For example, transitioning from in-person to telephone outcomes may alter the psychometric properties of a measure. In contrast, changes to the mode of data collection from paper to electronic case report forms are less likely to negatively impact internal validity, though this change should still be documented. Finally, pausing recruitment and enrolment to ensure participant and personnel safety may not have significant impacts on the scientific rigor of studies in progress, but will extend the duration of planned enrolment, and associated costs [16]. Notwithstanding, investigators will need to assess whether the trial management strategies implemented due to extenuating circumstances such as the COVID-19 pandemic affect the validity of trial results. For example, we collected 3 out of the 6 patients requiring primary outcome assessments. While our initial sample size calculation estimated that we needed to enroll 360 patients after accounting for ICU mortality and missing data, we plan to re-evaluate the effect of the pandemic in terms of the scope of any additional missing primary outcome data on the overall trial cohort as we develop our final statistical analysis plan.

The authors of CONSERVE 2021 define extenuating circumstances as, “Unavoidable situations that prompt modifications to a trial. These are not usually under the control of study investigators, sponsors, or funders.” [4] CONSERVE, which recommends reporting details about trial modifications, how the modifications are important, the potential impacts of modifications, and a timeline, suggests this extension *only* be used when the extenuating circumstances result in *important modifications* that could have a potentially meaningful effect on a study’s research question, ethics, internal validity and generalizability, feasibility, or analytical methods and statistical power [4]. In March 2020, the duration and impact of the circumstances imposed by the pandemic were unknown. To honor our commitment to enrolled patients and to continue to advance our research agenda in the midst of unknown circumstances, we recognized that a systematic evaluation of our study processes was needed to determine which, if any, would require modifications and how to effectively mitigate potential pandemic impacts. For example, we elected not to enroll patients with COVID-19 for infection control concerns and the possibility of transmitting the virus through the equipment; furthermore, strained therapy resources could impair trial fidelity. We focused on mitigating the impact of the pandemic to patients already enrolled in the trial. The pandemic pause in enrollment has resulted in delayed recruitment leading to a later trial closure date, although randomization has restarted. Clinically, given the emerging evidence on Long COVID outcomes, future studies of ICU rehabilitation in patients with COVID-19 will be critical, but this discussion is beyond the scope of this study.

**Fig. 5:**
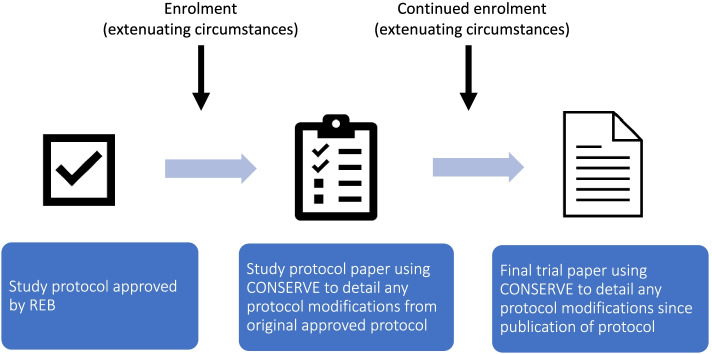
Depiction of the stages at which CONSERVE could be applied to a trial in progress

After careful evaluation, we did not make important modifications to the CYCLE intervention or outcomes assessments and conducted our interim analysis as planned. However, we did implement extensive mitigation strategies to protect participant flow and statistical analyses. For these reasons, we suggest the use of the CONSERVE reporting guidance for all trials that experienced extenuating circumstances as defined above—notably, all trials occurring during the COVID-19 pandemic. This is supported by CONSERVE authors [4] who state that while it was intended to capture important modifications, there may be opportunity to enhance reporting within a broader context. Understanding how trialists implement and evaluate mitigation strategies and their rationale for any important modifications will help future trialists respond and adjust to other unforeseen circumstances causing research disruptions. Given the known gap in evidence for trial management [17], the universal implementation of CONSERVE 2021 reporting in *all* instances of extenuating circumstances will enhance transparency in reporting and decision-making. In Fig. 5, we outline the stages in a study at which CONSERVE 2021 could be applied.

## Strengths and limitations

Strengths of this study include focus on the conduct of an international multi-center trial of a complex rehabilitation intervention in acute care during the pandemic. We addressed key issues of data integrity, data completeness, and research ethics using the CONSERVE 2021 statement as a reporting framework for our trial in-progress. We reported our specific Methods Centre processes in the context of the COVID-19 pandemic, including practical examples and resources which could be generalizable to other complex interventions or studies including remote telephone follow-up. Our report also has limitations. First, it is focused on one trial underway in the ICU setting with follow-up in-hospital and beyond. Second, it does not address other parts of CONSERVE 2021, such as changing recruitment strategies or statistical analysis plans due to extenuating circumstances.

## Conclusions

The pandemic has been a catalyst to identify guiding principles and develop contingency plans and mitigating strategies to optimize study recruitment, retention, fidelity and reporting in the face of unintended interruptions. Despite a complete pause in enrolment in this rehabilitation trial and an immediate transition to working from home for many research personnel, data integrity was maintained, the interim analysis was completed, and the study has since resumed enrolment at 10 of 15 sites.

## Supplementary Information

**Additional file 1: Appendix 1.** CONSERVE-CONSORT Extension checklist. **Appendix 2.** Site communication template. **Appendix 3.** Working From Home – Conducting CYCLE Follow-up Phone Calls Remotely. **Appendix 4.** Site restart communication template and planning template.

## Data Availability

The datasets used and/or analyzed during the current study are available from the corresponding author on reasonable request.

## Abbreviations

AUS: Australia
Ax: Assessment
CAN: Canada
CONSERVE: CONSORT and SPIRIT Extension for RCTs Revised in Extenuating Circumstances
CONSORT: Consolidated Standards of Reporting Trials
D: Day
D/C: Discharge
HCW: Healthcare worker
ICU: Intensive care unit
LTCHs: Long-term care homes
MC: Methods Centre
MV: Mechanical ventilation
ON: Ontario
PPE: Personal protective equipment
PR: Physical rehabilitation
PT: Physiotherapy
QC: Quebec
RC: Research coordinator
REB: Research Ethics Board
RCT: Randomized controlled trial
SDM: Substitute decision maker
SPIRIT: Standard Protocol Items: Recommendations for Interventional Trials
USA: United States of America
WHO: World Health Organization
