# Supplementary material for: Research interrupted: applying the CONSERVE 2021 Statement to a randomized trial of rehabilitation during critical illness affected by the COVID-19 pandemic
Source: Trials. 2022 Sep 2;23:735. doi: 10.1186/s13063-022-06640-y (PMC9438218; doi:10.1186/s13063-022-06640-y)
Supplement: Supplementary file 1 — Additional file 1: Appendix 1. CONSERVE-CONSORT Extension checklist. Appendix 2. Site communication template. Appendix 3. Working From Home – Conducting CYCLE Follow-up Phone Calls Remotely. Appendix 4. Site restart communication template and planning template. [file 13063_2022_6640_MOESM1_ESM.docx]

**Title:** Research interrupted: Applying the CONSERVE 2021 Statement to a randomized trial of rehabilitation during critical illness affected by the COVID-19 pandemic.

**Authors:**

Julie C Reid MScPT, PhD

Alex Molloy BSc

Geoff Strong MScPT, M.Binf

Laurel Kelly MScPT

Heather O’Grady BSc, PhD (c)

Deborah Cook MD, MSc

Patrick M Archambault MD, MSc

Ian Ball MD, MSc

Sue Berney PT, PhD

Karen EA Burns MD, MSc

Frederick D’Aragon MD, MSc

Erick Duan MD, MSc

Shane W English MD, MSc

François Lamontagne MD, MSc

Amy M Pastva PT, MA, PhD

Bram Rochwerg MD MSc

Andrew JE Seely MD, PhD

Karim Serri MD

Jennifer LY Tsang MD, PhD

Avelino C Verceles MD, MS

Brenda Reeve MD

Alison Fox-Robichaud MD, MSc

John Muscedere MD

Margaret Herridge MD, MSc, MPH

Lehana Thabane PhD

Michelle E Kho PT, PhD

On behalf of the CYCLE Investigators

**Additional File 1**

**(Appendices 1-4).**

# Appendix 1. CONSERVE-CONSORT Extension Checklist

| CONSERVE-CONSORT Extension: July 4, 2022 | | | | | |
| --- | --- | --- | --- | --- | --- |
| Item | Item Title | Description | | | Page No. |
| I. | Extenuating Circumstances | Describe the circumstances and how they constitute extenuating circumstances. | | | 6-7 |
| II. | Important Modifications | 1. Describe how the modifications are important modifications. | | | 8-9 |
|  |  | 1. Describe the impacts and mitigating strategies, including their rationale and implications for the trial. | | | (see below) |
|  |  | 1. Provide a modification timeline. | | | Figures 2, 3 |
| III. | Responsible Parties | State who planned, reviewed and approved the modifications. | | | 7 |
| IV. | Interim data | If modifications were informed by trial data, describe how the interim data were used, including whether they were examined by study group, and whether the individuals reviewing the data were blinded to the treatment allocation. | | | N/A |
| CONSORT Number and Item | | For each row, if important modifications occurred check “direct impact” and/or “mitigating strategy” and describe the changes in the trial manuscript or supplement. Check “no change” for items that are unaffected in the extenuating circumstance. | | | Page No. |
|  |  | No Change | Impact* | Mitigating Strategy** |  |
| 1 | Title and abstract | X |  |  |  |
| 2 | Introduction | X |  |  |  |
| 3 | Methods: Trial Design | X |  |  |  |
| 4 | Methods: Participants | X |  |  |  |
| 5 | Methods: Interventions | X |  |  |  |
| 6 | Methods: Outcomes | X |  |  |  |
| 7 | Methods: Sample Size | X |  |  |  |
| 8-10 | Methods: Randomisation | X |  |  |  |
| 11 | Methods: Blinding | X |  |  |  |
| 12 | Methods: Statistical methods |  | Page 9 | Page 12 |  |
| 13 | Results: Participant flow |  | Page 7 | Pages 7, 8, 11 |  |
| 14 | Results: Recruitment |  | Page 13 | Pages 8, 13 |  |
| 15 | Results: Baseline data | X |  |  |  |
| 16 | Results: Numbers analysed | X |  |  |  |
| 17 | Results: Outcomes and estimation | X |  |  |  |
| 18 | Results: Ancillary analyses | X |  |  |  |
| 19 | Results: Harms | X |  |  |  |
| 20 | Discussion: Limitations | X |  |  |  |
| 21 | Discussion: Generalisability | X |  |  |  |
| 23 | Other information: Registration | X |  |  |  |
| 24 | Other information: Protocol | X |  |  |  |
| 25 | Other information: Funding | X |  |  |  |
| *Aspects of the trial that are directly affected or changed by the extenuating circumstance and are not under the control of investigators, sponsor or funder.  **Aspects of the trial that are modified by the study investigators, sponsor or funder to respond to the extenuating circumstance or manage the direct impacts on the trial. | | | | | |

**Appendix 2.** Site communication template

Hi [RC name(s)],

We hope you are staying well and safe in this unprecedented situation.

I am reaching out on behalf of the CYCLE Methods Centre to ask a couple of questions. We appreciate these are challenging times and many non-clinical staff are being asked to work remotely. With that in mind, we are trying to determine the following (not all of these will be applicable, please see below for a site-specific summary):

**a)** the **status of CYCLE patients still in hospital** and plan for collecting any remaining PT and/or RC assessments (please don't hesitate to reach out if we can help you develop strategies for coordination and collection) -

*-from our records, there are no CYCLE patients still in hospital*

**b)** the **status of, and/or plan for, collecting 90-day assessments** for those patients that are due in the next few weeks -

*-from our records, there is 1 patient with a past-due 90-day assessment:*

*patient 4, 90-day window Dec. 15 - Jan. 21 - may we please ask for an update on the status of this assessment?*

**c)** does your site have**research staff that are continuing to work**(either on site or remotely) that could work on data entry and cleaning for CYCLE patients (with access to medical records, CRFs, and iDataFax)?

The following are the**patients we are prioritizing for data entry and validation**, as they will contribute to the 180-patient interim analysis: *patients 1, 3, 4*

Given the fluid nature of this situation and the quickly changing landscape, could we please ask you to keep us up-to-date on any major changes that may impact any of the above?

Thanks very much for your continued support. Please feel free to reach out if there's anything we can assist with. Stay safe and warm regards,

**Appendix 3.** Working from Home – conducting CYCLE follow-up phone calls remotely
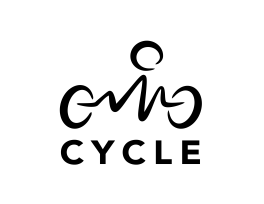


1. Patient Contact Information

- If it is possible to obtain the patient’s name and phone number by accessing his or her electronic medical record from home this method is recommended as it can be done securely
- If you must access written or electronic data to obtain this information, please ensure you are storing this identifying information in a secure manner (i.e., password protected and encrypted files, private cabinet) in order to maintain patient confidentiality

2. Blocking Outgoing Caller Identification

- To maintain your privacy when conducting follow-up phone calls from your personal device, please hide your caller identification. There are many ways to do this, depending on the device:

How to hide caller ID on your **iPhone**:

1. From the home screen, tap **Settings**
2. Tap the **Phone** icon
3. Tap **Show My Caller ID**
4. Toggle the button to ‘off’ (bubble should be on the left)

How to hide caller ID on your **Samsung Galaxy**:

1. From the home screen, tap the **Phone** icon
2. Tap the **Menu** icon (⋮)
3. Tap **Settings**
4. Tap **Supplementary Services**
5. Tap **Show My Caller ID**
6. Tap your caller ID preference to hide your phone number

How to hide caller ID on your **Android** phone:

1. Open the **Phone** app
2. Open the **Menu**
3. Select **Settings**
4. Click on **Call Settings**
5. Click on **Additional Settings**
6. Click on **Caller ID**
7. Choose **Hide number**

Please note: If the above steps do not work, Google “*how to hide phone number*” followed by your phone make and model.

**Additional ways to hide caller ID:**

To hide your cell phone number for a single call, enter **#31#** followed by the number. Your caller ID will show up as “Private Caller”.

To hide your landline phone number for a single call, enter ***67** followed by the number.

3. Leaving a Message

- If you need to leave a message, we recommend leaving the patient with your office phone number. Let them know that you will not be answering this phone but will be checking the messages periodically. Ask them to leave a message stating the best time/date to return their call. You can then access your office voice mail remotely to retrieve this information.

4. Call History

- To maintain confidentiality of patient contact information, please delete the call history from your mobile device immediately after completing the call.

5. Data Storage

- The 90-day follow-up assessment does not require the collection of any identifying information. Please ensure you are not recording any personal identifiers on this form. The forms should still be stored in a secure manner in order to maintain participant privacy and confidentiality.

Please follow your institution’s privacy department directives for maintaining patient privacy and confidentiality while working from home.

**Questions?**

Please contact the CYCLE Methods Centre if you have any questions regarding remotely conducting CYCLE follow-up phone calls.

| **[Methods Centre RC]**  Email:  Cell: | **[Methods Centre RC]**  Email: |
| --- | --- |

**Appendix 4.** Site restart communication template and planning template

Hi [RC name(s)],

Thank you for all your help with CYCLE, especially with data entry and responding to queries as we prepare for the interim analysis. Two weeks ago, we received news from both McMaster University and the Research Institute of St. Joseph’s (SJH), that clinical research can resume.  To date, 6 sites including [site], [site], [site], [site], [site], [site] have re-started CYCLE.

Is [specific site] open to re-starting CYCLE? We understand that not all sites will be ready to resume, and your institution may not yet have provided guidance for re-starting clinical research in the ICU. However, we would like to use this opportunity to start the discussion.

In anticipation of resuming CYCLE at [CYCLE home site], we developed a plan to prepare for screening, delivering care, and assessing outcomes.  During the earlier part of the pandemic, we had almost half of our physio department off work due to COVID cases and contacts. This would have had a major impact if we had a patient on study, so we are asking sites to develop similar plans in the event that COVID disrupts research again. To help sites think through the re-start, we are attaching a work sheet and we ask you to please submit your plans and back-up plans for cycling and assessments for each time point. For your reference, we are attaching the work sheet that was developed at [CYCLE home site].

Could you please send us a copy of the work sheet for [specific site] before re-starting? Please do not hesitate to reach out with any questions or concerns.

Thanks for considering and we look forward to having your site back up and running.

**Planning for [site] CYCLE restart as of [date], 2020**

Awake

Test #1

**Clinical Course**

**Study Outcome Assessments**

ICU Admission

Randomized/ Study Entry ≤4 d MV

ICU Discharge

Test #2

Test #4

3 d Post-ICU Discharge

Test #3

30 min Cycling + Routine PT *or* Routine PT

Intubated

Hospital Discharge

90 Day Post Randomization

Routine PT*

Test #5

|  | **ICU Intervention** | **ICU Awakening** | **ICU Discharge** | **3d post ICU Discharge**** | **Hospital Discharge** | **90d post Randomization Telephone** |
| --- | --- | --- | --- | --- | --- | --- |
| Plan |  | S+F:  IPAT: | S+F:  RC: | S+F: | S+F:  RC: |  |
| 1^st^ backup |  | S+F:  IPAT: | S+F:  RC: | S+F: | S+F:  RC: |  |
| 2^nd^ backup |  | S+F:  IPAT: | S+F:  RC: | S+F: | S+F:  RC: |  |

**Legend:** * routine PT continues throughout the hospital stay until discharge; ** primary outcome.

**Abbreviations:** ICU – intensive care unit; d – day; MV – mechanical ventilation; PT – physiotherapy; S + F – strength and function; IPAT – Intensive Care Psychological Assessment Tool; RC – research coordinator.

**Outcome Assessors**

| **Name** | **Blinding Status** | **Unit** | **Notes** |
| --- | --- | --- | --- |
|  | Unblinded |  |  |
|  | Unblinded |  |  |
|  | Unblinded |  |  |
|  | Unblinded |  |  |
|  | Unblinded |  |  |
|  | Blinded |  |  |
|  | Blinded |  |  |
|  | Blinded |  |  |
|  | Blinded |  |  |
|  | Blinded |  |  |
|  | Blinded |  |  |
|  | Blinded |  |  |
|  | Blinded |  |  |

**Other Considerations:**Screening/Consent

**- We will not enroll patients with COVID at this time**
- **Research Coordinators**: Screen patient charts for COVID testing, signs/symptoms of COVID, discuss potential of COVID with team and avoid enrolling patients with suspicion for COVID (temp exemption “other”)
- Try to wait until later in 4-day inclusion window to enroll if possible to ensure patient not COVID positive if suspected COVID

Interventions in ICU
- If study patient becomes COVID positive: no change for routine PT – assess patient for appropriateness for Rx as normal. If patient randomized to cycling, then bike not to be used with other patients until the study patient is discharged from ICU >72 hrs

Outcome Assessments
- Main goal: minimize potential exposures and conserve PPE
- Offer existing blinded assessors first opportunity to re-engage in the study
- Leave tub of Virox in assessment equipment bag for cleaning after use

Guidelines for Minimizing Exposure/Travel between units (St. Joe’s Physiotherapy policies; apply local policies as applicable)
- Guidance to therapists: avoid travel between units multiple times per day
- Ideally 2 units/day. “Clean” units then to units where care for patients with COVID occurs (ICU, CCU, COVID unit, COVID warm unit)
- Plan for assessors from non-COVID units to perform assessments in non-COVID areas, and assessors from COVID units to perform assessments in COVID areas if needed.
